# Increased levels of circulating microparticles in primary Sjögren's syndrome, systemic lupus erythematosus and rheumatoid arthritis and relation with disease activity

**DOI:** 10.1186/ar2833

**Published:** 2009-10-15

**Authors:** Jérémie Sellam, Valérie Proulle, Astrid Jüngel, Marc Ittah, Corinne Miceli Richard, Jacques-Eric Gottenberg, Florence Toti, Joelle Benessiano, Steffen Gay, Jean-Marie Freyssinet, Xavier Mariette

**Affiliations:** 1 Rhumatologie, Hôpital Bicêtre, Assistance Publique-Hôpitaux de Paris (AP-HP), INSERM U802, Université Paris-Sud 11, 78 rue du Général Leclerc, 94270, Le Kremlin Bicêtre, France; 2 Hématologie, Hôpital Bicêtre, APHP, INSERM U770, Université Paris-Sud 11, 78 rue du Général Leclerc, 94270, Le Kremlin Bicêtre, France; 3 Center of Experimental Rheumatology, University Hospital Zurich, Gloriastrasse 25, CH 8091 Zurich, Switzerland; 4 INSERM Unité 770 et Université de Strasbourg, 78 rue du Général Leclerc, 94270, Le Kremlin Bicêtre, France; 5 Centre de Ressources biologiques - Centre d'Investigation clinique, Hôpital Bichat, AP-HP, 46, rue Henri-Huchard, 75018 Paris, France

## Abstract

**Introduction:**

Cell stimulation leads to the shedding of phosphatidylserine (PS)-rich microparticles (MPs). Because autoimmune diseases (AIDs) are characterized by cell activation, we investigated level of circulating MPs as a possible biomarker in primary Sjögren's syndrome (pSS), systemic lupus erythematosus (SLE) and rheumatoid arthritis (RA).

**Methods:**

We measured plasma levels of total, platelet and leukocyte MPs by prothrombinase capture assay and flow cytometry in 43 patients with pSS, 20 with SLE and 24 with RA and in 44 healthy controls (HCs). Secretory phospholipase A2 (sPLA2) activity was assessed by fluorometry. Soluble CD40 ligand (sCD40L) and soluble P-selectin (sCD62P), reflecting platelet activation, were measured by ELISA.

**Results:**

Patients with pSS showed increased plasma level of total MPs (mean ± SEM 8.49 ± 1.14 nM PS equivalent (Eq), *P *< 0.0001), as did patients with RA (7.23 ± 1.05 n PS Eq, *P *= 0.004) and SLE (7.3 ± 1.25 nM PS Eq, *P *= 0.0004), as compared with HCs (4.13 ± 0.2 nM PS Eq). Patients with AIDs all showed increased level of platelet MPs (*P *< 0.0001), but only those with pSS showed increased level of leukocyte MPs (*P *< 0.0001). Results by capture assay and flow cytometry were correlated. In patients with high disease activity according to extra-glandular complications (pSS), DAS28 (RA) or SLEDAI (SLE) compared with low-activity patients, the MP level was only slightly increased in comparison with those having a low disease activity. Platelet MP level was inversely correlated with anti-DNA antibody level in SLE (r = -0.65; *P *= 0.003) and serum β2 microglobulin level in pSS (r = -0.37; *P *< 0.03). The levels of total and platelet MPs were inversely correlated with sPLA2 activity (r = -0.37, *P *= 0.0007; r = -0.36, *P *= 0.002, respectively). sCD40L and sCD62P concentrations were significantly higher in pSS than in HC (*P *≤ 0.006).

**Conclusions:**

Plasma MP level is elevated in pSS, as well as in SLE and RA, and could be used as a biomarker reflecting systemic cell activation. Level of leukocyte-derived MPs is increased in pSS only. The MP level is low in case of more severe AID, probably because of high secretory phospholipase A2 (sPLA2) activity, which leads to consumption of MPs. Increase of platelet-derived MPs, sCD40L and sCD62P, highlights platelet activation in pSS.

## Introduction

A general feature of activated cells is their ability to shed fragments from their plasma membrane. These fragments represent a heterogeneous population of small membrane-coated vesicles with diameter of 0.1 to 1 μm, termed microparticles (MPs) [1]. MPs belong to the family of circulating vesicles, including apoptotic bodies and exosomes, and can be detected in all biological fluids, especially plasma. MPs have to be differentiated from exosomes and from apoptotic bodies. Exosomes are smaller than MPs and not generated from the plasma membrane but arise from the inside of cells in multivesicular bodies, and are mostly devoid of phosphatidylserine. Apoptotic bodies are formed during the final stages of programmed cell death and are generally larger in diameter and volume than MPs [1]. The outer layer of the bilayer membrane of MPs contains aminophospholipids, mainly anionic phosphatidylserine (PS), which is procoagulant and detectable by its binding to annexin V. MPs also contain protein markers specific to the parental cell types, which allows for the detection of the cellular origin of MPs [2]. These subcellular structures can transfer bioactive molecules from parental to target cells, thus allowing for regulation and amplification of several biological mechanisms such as apoptosis or cell activation (inflammatory or autoimmune responses, cell proliferation or coagulation). Hence, MPs could reflect parental cell stimulation and be involved in target cell stimulation [2].

Because of these properties, MPs have been associated with systemic inflammation or excessive risk of thrombosis in various diseases, such as rheumatoid arthritis (RA), systemic lupus erythematosus (SLE), vasculitis and antiphospholipid syndrome (APLS).

Similar to RA and SLE, primary Sjögren's syndrome (pSS) is an autoimmune disease (AID) characterized by leukocyte activation. Platelet activation has been reported in SLE and RA, but this feature, illustrated by increased level of plasma soluble CD40 ligand (sCD40L), has been noted only once in pSS [3,4].

We aimed to assess the plasma level of annexin V-positive (e.g., PS-positive or total), leukocyte and platelet circulating MPs in pSS and other AIDs (SLE and RA) as a biomarker of cell activation.

## Materials and methods

### Materials and controls

The characteristics of all subjects are shown in Table 1. We obtained blood samples from 43 patients with pSS fulfilling American-European Consensus Group criteria [5], 20 with SLE fulfilling American College of Rheumatology criteria [6] and 24 with RA fulfilling American College of Rheumatology criteria [7] in the Department of Rheumatology of Bicêtre University Hospital. The study was approved by the local research ethics committee, and informed written consent was obtained from all patients.

**Table 1:** Characteristics of subjects

	pSS	SLE	RA	HC
Number of patients	43	20	26	44
Sex, male/female	1/42	1/19	5/21	7/37
Age, median (range)	60 (26-77)	35.5 (26-77)	55 (23-81)	41 (19-65)
Disease duration, median (range)	9 (1-22)	6 (0.5-31)	5.5 (0.5-41)	NA
Positive anti-Ro(SSA) Ab, n (%)	32 (74)	NA	NA	NA
Positive anti-La(SSB) Ab, n (%)	17 (39)	NA	NA	NA
Focus score ≥ 1, n (%)	39 (91)	NA	NA	NA
Extraglandular manifestations, n (%)	17 (36)	NA	NA	NA
Positive anti-DNA antibody, n (%)	NA	16 (84%)	NA	NA
Positive anti-CCP antibody, n (%)	NA	NA	23 (88)	NA
Positive rheumatoid factor, n (%)	NA	NA	24 (92)	NA
Malignant hemopathy, n (%)	5 (12)	0 (0)	0 (0)	0 (0)
SLEDAI, median (range)	NA	3 (1-12)	NA	NA
DAS28, median (range)	NA	NA	4.8 (1.2-6.3)	NA
Secondary APLS	0	4	0	NA
ESR (mm), median (range)	22 (4-44)	22 (4-48)	25 (4-104)	NA
C-reactive protein (mg/L), median (range)	1.0 (1-11)	1.5 (1-11)	6 (1-106)	NA
Fibrinogen (g/L), median (range)	3.2 (2.6-4.5)	3.4 (2.2-4.8)	4.4 (2.4-8.6)	2.9 (1.7-4.5)
Beta2 microglobulin level (mg/L), median (range)	2.4 (1.6-5.6)	NA	NA	NA
Immunosuppressive drug use, n (%)	7 (16)	11 (55)	19 (73)	NA

Ab = antibody; APLS = anti-phospholipid syndrome; CCP = cyclic citrullinated peptide; DAS28 = Disease Activity Score for 28 Joints; ESR = erythrocyte sedimentation rate; HC = healthy control; NA = not applicable; pSS = primary Sjogren's syndrome; RA = rheumatoid arthritis; SLE = systemic lupus erythematosus; SLEDAI = SLE disease activity score.

Among the 43 pSS patients, extra-glandular involvement as previously defined [8] was present in 17 patients: lung involvement (n = 3), neurological involvement (n = 4), active synovitis (n = 2), myositis (n = 2), vasculitis (n = 2), renal involvement (n = 1), and lymph node enlargement (n = 3). Five patients had malignant hemopathy, three with marginal zone lymphoma (one current, two previous) and two current multiple myeloma, and two had monoclonal gammopathy of undetermined significance (MGUS). Seven pSS patients received immunosuppressive drugs (rituximab, n = 3; rituximab plus methotrexate, n = 1; cyclophosphamide plus melphalan, n = 1; methotrexate, n = 2).

For patients with SLE, disease activity was measured by the SLE Disease Activity Index (SLEDAI) on the day of blood testing [9]. Eleven patients received immunosuppressive drugs (mycophenolate mofetyl, n = 7; azathioprine, n = 2; rituximab, n = 2; prednisone >10 mg daily, n = 4). Four patients presented with a secondary anti-phospholipid syndrome according to international criteria [10]. Patients with acute or chronic infections or with primary anti-phospholipid syndrome were excluded from the study.

For RA patients, disease activity was measured by the Disease Activity Score for 28 joints (DAS28) on the day of blood testing. Immunosuppressive agents were given to 19 RA patients (methotrexate, n = 16; anti-TNFα agents, n = 5; leflunomide, n = 2); no patient received steroids more than 10 mg daily.

As controls, after informed consent was obtained, we used a group of 44 healthy controls (HCs) who presented no inflammatory, neoplasic, autoimmune or metabolic diseases.

Cardiovascular risk factors (diabetes, smoking, arterial hypertension, hyperlipidemia) were noted in three patients with pSS, one with SLE, and six with RA. Of note, at the time of blood testing, no patient or controls presented signs of acute thrombosis or infection known to modify the plasma level of MPs.

### MP isolation from plasma

According to a standardized procedure [11,12], after collection of citrated fresh blood samples, MPs were isolated by double centrifugation at 1500 *g *for 15 minutes and 13,000 *g *for 2 minutes at room temperature and immediately stored at -80°C for further analysis. This procedure has been previously validated as mainly yielding MPs and excluding larger apoptotic bodies, eliminated by the two centrifugation steps [12].

An aliquot of plasma obtained before the second centrifugation was kept for assessment of secretory phospholipase A2 (sPLA2) activity and sCD40L and soluble P-selectin (sCD62P) content.

### MP quantification by functional prothrombinase capture assay

Circulating MPs were captured onto insolubilized annexin V and were called total MPs because annexin V-positive MPs represent the large majority of the MP population. Capture with monoclonal antibodies (mAbs) (mAb against human platelet anti-glycoprotein Ib (GPIb) and CD11a) was performed for platelet and leukocyte MP isolation, respectively. Then quantification of these captured MPs was performed with a functional prothrombinase assay in which concentrations of purified clotting factors and calcium (factor Xa, factor Va, prothrombin and CaCl_2_) were determined to ensure that PS was the rate-limiting parameter of the generation of thrombin from prothrombin [11,12]. Thus, thrombin generation is dependant on the PS content, which is proportional to the immobilized MPs. Results are expressed as nanomolar PS equivalent (nM PS Eq) by reference to a standard curve constructed with liposomes of known PS concentrations [13]. For capture by CD11a and GPIb, background values obtained with irrelevant immunoglobulin (Ig) Gs of corresponding isotypes were subtracted from those measured with specific mAbs. Different affinities of MPs for annexin V and mAbs prevent direct comparison or addition between levels of MPs measured with use of these ligands.

### MP quantification by flow cytometry

Flow cytometry experiments were adapted from Combes and colleagues [14] and Robert and colleagues [15]. All analyses were performed by use of a fluorescent-activated cell sorter (FACS; EPICS XL; Beckman Coulter, Roissy, France) and RXP-software analysis (Beckman Coulter). Forward scatter and side scatter were set as a logarithmic gain, and Megamix (Biocytex, Marseille, France), containing a mix of fluorescent microbeads of various diameters (0.5, 0.9 and 3.0 μm), was used for initial settings and before each experiment to measure MPs, as an internal control. Gates were then set to include events between 0.5 and 1.0 μm with exclusion of background corresponding to debris usually present in buffers.

We incubated 40 μL of platelet-free plasma MPs for 30 minutes in the dark at room temperature with annexin V-fluorescein isothiocyanate (FITC; Beckman Coulter, Roissy, France), specific antibodies or isotype-matched irrelevant control (10 μL) conjugated with phycoerythrin after gentle shaking, and 400 μL of annexin V buffer (containing calcium ions) or PBS1X was added before immediate acquisition. Two negative controls of annexin V ligation were used: MPs incubated with annexin V in a calcium-free buffer (to prevent annexin V ligation to PS) or without annexin V in a specific annexin V buffer to estimate the auto-fluorescence.

MP subpopulations were determined according to the expression of membrane-specific antigens from platelets and leukocytes by use of anti-CD61 and anti-CD45 mAbs (Beckman Coulter, Roissy, France), respectively. Staining with isotype-matched irrelevant mAbs (Beckman Coulter, Roissy, France) at the same concentration and under the same conditions was used as a control.

Before acquisition, a known number of 3 μm calibrated microbeads (Sigma Aldrich, Saint Louis, MO, USA) was placed in each tube and run concurrently with the MP samples in the FACS, thus allowing for quantitative determination of MPs (annexin V-positive or from different origin). The absolute number of MPs per millimeter plasma was then determined by counting the proportion of beads and the exact volume of plasma from which MPs were analyzed. The analysis was stopped when a fixed number of microbeads (10,000) were counted. Results are expressed as number of MPs per microliter by the formula N = (MP × beads per tube/volume of plasma)/number of counted beads.

### Measurement of sPLA2 activity and sCD40L and sCD62P content in plasma

We assessed the functional activity of sPLA2 because plasma sPLA2 is able to hydrolyze phospholipids such as PS or phosphatidylcholine present in MPs [16]. Plasma sPLA2 activity, expressed as nanomoles per minute per millilitre (nmol/min per mL), was measured by selective fluorometric assay as previously described [17].

Platelet activation was assessed by measurement of sCD40L and sCD62P concentrations in plasma by use of the human sCD40L Quantikine kit and a human sCD62P Immunoassay (R&D Systems, Lille, France), respectively, following the manufacturer's instructions.

### Other biological parameters

Anti-Ro/SSA and anti-La/SSB antibodies and IgG anti-double-stranded DNA (dsDNA) antibodies were determined by counter-immunoelectrophoresis and ELISA, respectively, as described previously [18]. The serum β2 microglobulin level was determined by nephelometry (Array 360 system, Beckman Coulter, Roissy, France) as previously described [8].

For biological anti-phospholipid investigations, anticardiolipin and anti-β2GPI antibody levels were assessed by ELISA (Biorad, Marne la Coquette, France and INOVA Diagnostics, San Diego, CA, USA, respectively).

Other biological tests were performed as routine in the Departments of Hematology and Biochemistry of our hospital (leukocyte and platelet counts, fibrinogen and C-reactive protein levels).

### Statistical analysis

Characteristics of patients are expressed as number and percentage and median and range. Results for MP levels are expressed as mean ± standard error of the mean. Comparisons of mean MP levels, sPLA2 activity, sCD40L and sCD62P concentrations between different groups of subjects (independent analysis) were analyzed by non-parametric Mann-Whitney U test. Spearman's rank correlation coefficients were calculated to investigate the relation between MP counts and clinical and biological parameters. A *P *< 0.05 was considered statistically significant. Statistical analysis involved use of GraphPad Prism 5 software (GraphPad Software Inc., San Diego, CA, USA).

## Results

### Measurement of circulating MPs in pSS and other AIDs by capture assay

**Figure 1:**
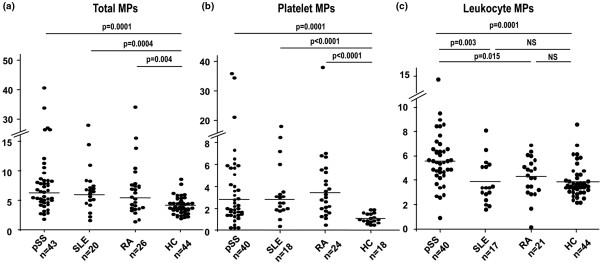
Plasma level of circulating microparticles. **(a) **Total microparticles (MPs); **(b) **platelet-derived MPs; **(c) **leukocyte-derived MPs in patients with primary Sjögren's syndrome (pSS), systemic lupus erythematosus (SLE), rheumatoid arthritis (RA) and healthy controls (HCs) by solid-phase capture with functional prothrombinase assay. Results are expressed as nM PS Eq. Horizontal lines show the mean value. Differences between groups were analyzed by the Mann-Whitney U test. All comparisons not specified in the figure were not significant (NS).

MPs detectable by capture onto annexin V were measured in 43 pSS, 20 SLE and 26 RA patients and 44 HCs. The level of total MPs was significantly higher in patients with pSS (8.49 ± 1.14 nM PS Eq), SLE (7.3 ± 1.25 nM PS Eq), and RA (7.23 ± 1.05 nM PS Eq) than in HCs (4.13 ± 0.2, *P *< 0.004; Table 2, Figure 1a). This increase involved particularly platelet-derived MPs (Table 2, Figure 1b). However, pSS, SLE and RA patients did not differ in level of total or platelet MPs.

**Table 2:** Level of circulating microparticles (MPs), secretory phospholipase A2 (sPLA2), sCD40L, sCD62P in patients with primary Sjögren syndrome (pSS), systemic lupus erythematosus (SLE), rheumatoid arthritis (RA) and in healthy controls (HCs)

		pSS	SLE	RA	HC
**MP level by capture assay, nM PS Eq**	**Total MPs (n)**	8.49 ± 1.14 (43)	7.3 ± 1.25 (20)	7.23 ± 1.05 (26)	4.13 ± 0.2 (44)
	**Platelet GPIb+ MPs (n)**	4.89 ± 1.25 (40)	4.28 ± 0.36 (18)	4.86 ± 1.48 (24)	1.12 ± 0.11 (18)
	**Leukocyte CD11a+ MPs (n)**	5.78 ± 0.37 (40)	3.89 ± 0.4 (17)	4.28 ± 0.9 (21)	3.92 ± 0.21 (44)

**MP number/μL plasma by FACS**	**Total MPs (n)**	91,700 ± 31,292 (5)	71,230 ± 19,160 (4)	127,200 ± 46,825 (2)	6422 ± 3472 (5)
	**Platelet CD61+ MPs (n)**	48,930 ± 18,260 (4)	32,290 ± 17,250 (4)	94370 ± 46,584 (2)	4229 ± 3914 (4)
	**Leukocyte CD45+ MPs (n)**	927 ± 729 (4)	422 ± 149 (4)	304 ± 33 (2)	190 ± 100 (4)

**sPLA2 activity, nmol/min/mL (n)**		50.9 ± 3.5 (37)	60.7 ± 8.0 (17)	69.8 ± 9.3 (25)	41.8 ± 3.4 (28)
**sCD40L, pg/mL (n)**		233 ± 31.7 (33)	262.6 ± 63.9 (17)	345.7 ± 67.23 (25)	133.6 ± 4.8 (33)
**sCD62P, ng/mL (n)**		38.9 ± 2.5 (34)	39.3 ± 3.7 (16)	43.4 ± 3.0 (25)	23.0 ± 1.0 (32)

Results are expressed as mean ± standard error of the mean. The number of patients tested is indicated in each box (n).

The level of leukocyte-derived MPs was higher in patients with pSS than in HCs (5.78 ± 0.37 versus 3.92 ± 0.21 nM PS Eq, *P *< 0.0001), with no difference between HCs and patients with SLE or RA (3.89 ± 0.4 and 4.28 ± 0.9, *P *= 0.46 and *P *= 0.18, respectively; Table 2, Figure 1c). Moreover, the level of leukocyte-derived MPs in pSS was significantly higher than that in SLE or RA (*P *= 0.003 and *P *= 0.015, respectively; Figure 1c).

The number of patients with cardiovascular comorbidities was low, yet after excluding these subjects, the results of statistical analyses remained unchanged (data not shown). Moreover, in pSS patients, the MP levels was the same in patients with hemopathy (lymphoma, multiple myeloma or MGUS; n = 7) and the others (n = 36; total MPs: 8.6 ± 1.3 vs 7,8 ± 2, *P *= 0.93). Likewise, MPs level was not different in SLE patients with (n = 4) or without secondary APLS (n = 16; total MPs: 7.7 ± 1.3 vs 5.5 ± 1.2, *P *= 0.48).

Of note, the level of leukocyte-derived MPs and absolute leukocyte count were not correlated, nor was the level of platelet MPs and platelet count in each group of patients correlated (data not shown).

### Flow cytometry measurement of circulating MPs

**Figure 2:**
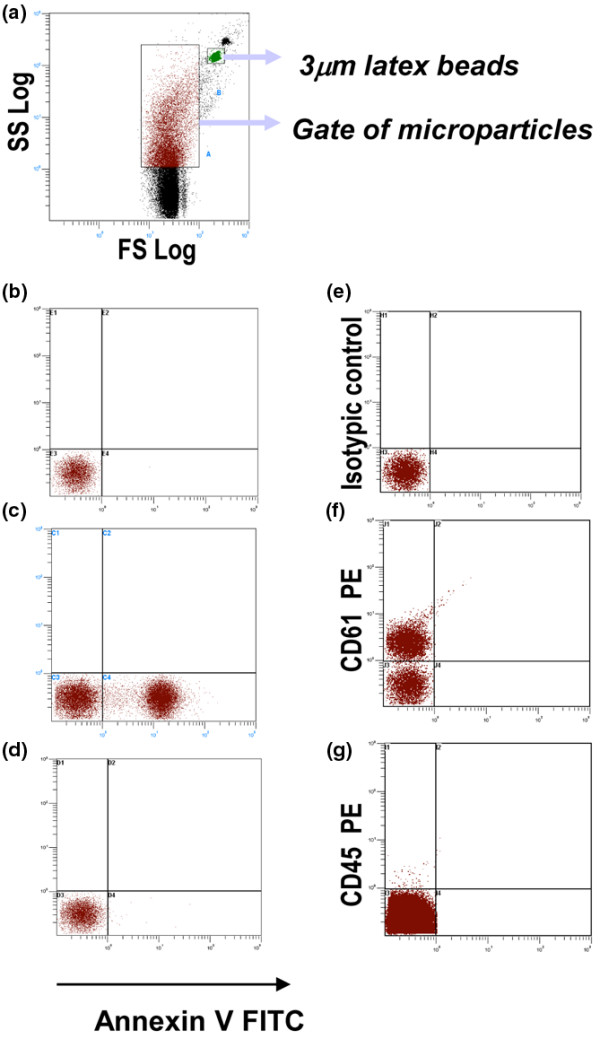
Representative flow cytometry density plots showing the gating protocol for microparticles. The gate of microparticles (MPs) was defined by use of Megamix containing fluorescent latex microbeads (0.5, 0.9 and 3 μm) **(a) **Quantitative estimation of MPs involved use of a fixed number of 3 μm microbeads, which were counted concomitantly with MP acquisition in the specific gate. **(b to d)** Gated MPs alone (b) without annexin V addition, (c) stained with annexin V FITC in a calcium-specific buffer, and (d) stained with annexin V in PBS (without calcium). **(e) **Isotype controls, **(f) **platelet MPs (CD61+), **(g) **leukocytes MPs (CD45+) using a single staining.

We assessed the plasma levels of total, leukocyte and platelet MPs by concomitant capture assay and flow cytometry in 17, 8 and 15 subjects, respectively. Results are in Table 2, and a representative staining is in Figure 2. Results from the two measurements showed a significant positive correlation for total MPs (r = 0.72, *P *= 0.001) as well as for platelet MPs (r = 0.76, *P *= 0.04) and for leukocyte MPs (r = 0.54, *P *= 0.04).

### Correlation of MP level with disease activity of pSS and other AIDs

**Figure 3:**
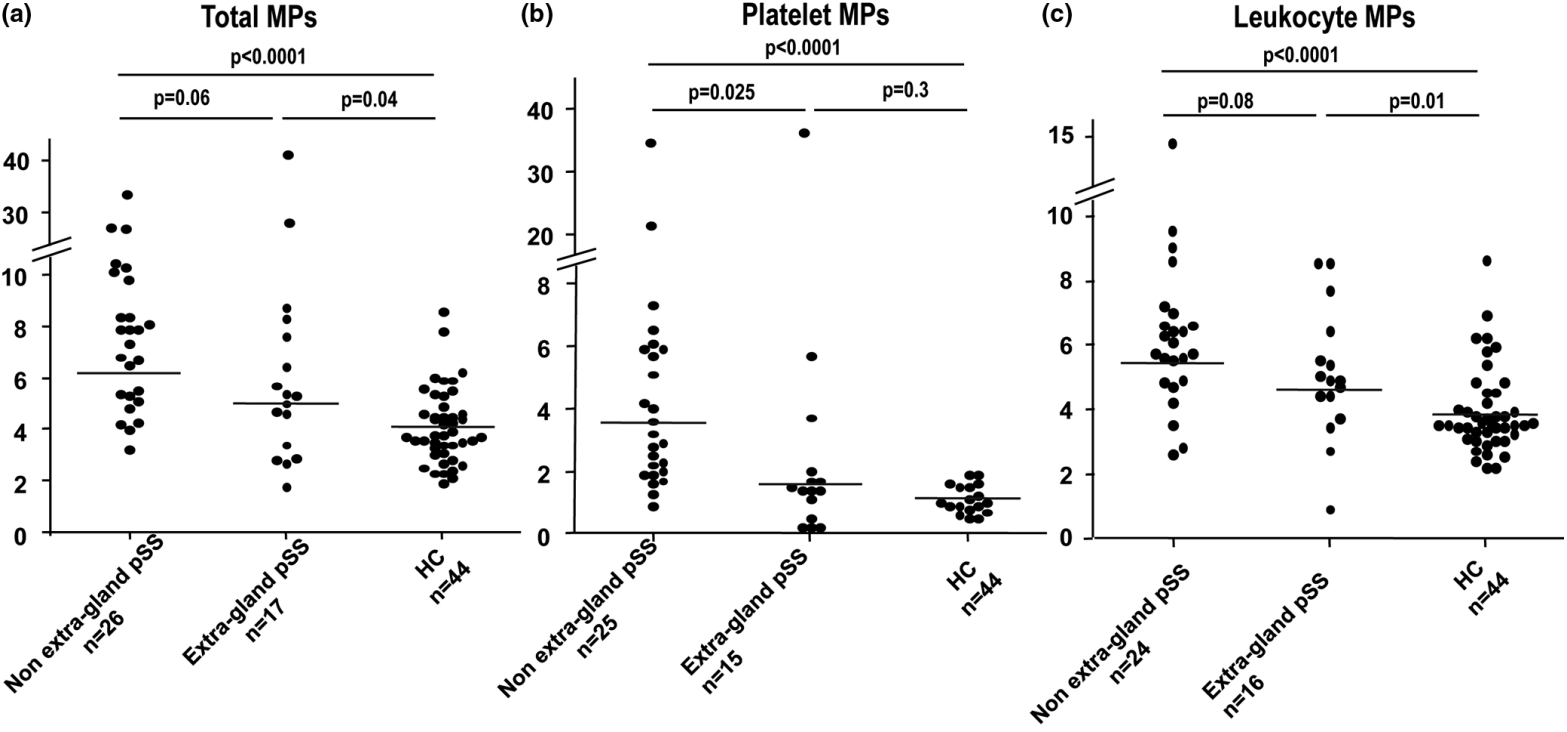
Plasma level of circulating microparticles. **(a) **Total microparticles (MPs); **(b) **platelet-derived MPs; **(c) **leukocyte-derived MPs in patients with primary Sjögren's syndrome (pSS) according to presence or not of extra-glandular involvement and in healthy controls (HCs) by solid-phase capture associated with functional prothrombinase assay. Results are expressed as nM PS Eq. Solid bars show the mean.

As MPs can reflect the state of cellular stimulation, we hypothesized that the level of MPs could be associated with AID activity. Platelet MP levels were significantly lower in pSS patients with extra-glandular involvement (3.88 ± 2.3 nM PS Eq) than in those with only glandular involvement (5.5 ± 1.45 nM PS Eq, *P *= 0.02) with a similar tendency for total (7.93 ± 2.25 versus 8.86 ± 1.2 nM PS Eq, *P *= 0.06) and leukocyte MPs (5.06 ± 0.5 versus 6.25 ± 0.5 nM PS Eq, *P *= 0.08; Figure 3).

**Figure 4:**
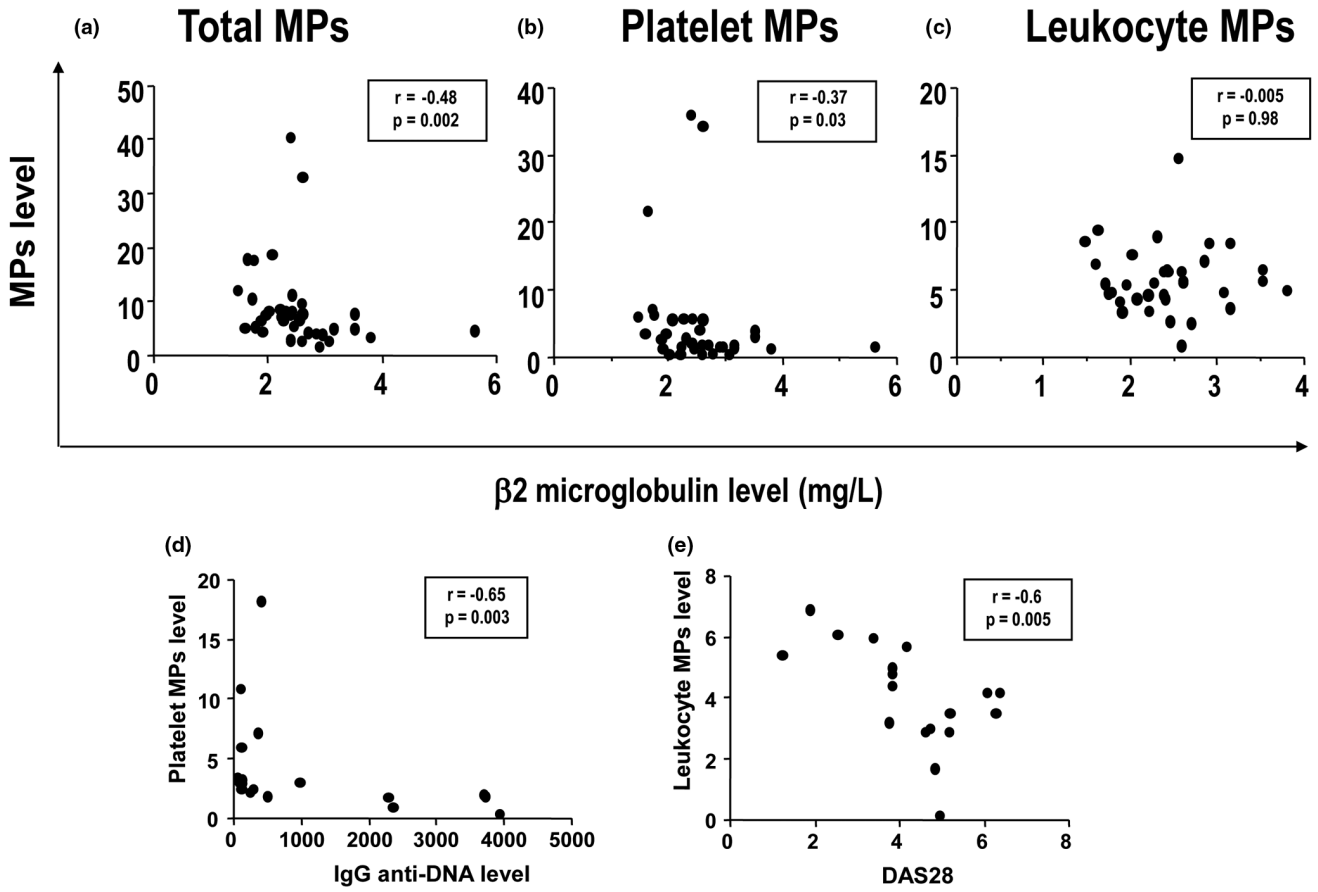
Correlation between serum level of beta 2 microglobulin (mg/L) and plasma level of total microparticles. **(a) **Platelet-derived microparticles (MPs), and **(c) **leukocyte-derived MPs (nM PS Eq) in primary Sjögren's syndrome (pSS). **(d) **Correlation between level of IgG anti-double-stranded DNA antibody (IU/L) and platelet MPs in SLE patients. **(e) **Correlation between disease activity score 28 (DAS28) and leukocyte MPs in RA patients.

Serum β2 microglobulin level, a B-cell activation marker associated with extra-glandular involvement [8], was inversely correlated with level of annexin V-positive MPs (r = -0.48, *P *= 0.002) and platelet MPs (r = -0.37, *P *= 0.03; Figures 4a to 4c).

Interestingly, we found similar results for SLE patients: a significant negative correlation between level of platelet MPs and level of anti-double-stranded DNA IgG (r = -0.65, *P *= 0.003; Figure 4d) and a negative correlation, although not significant, between level of platelet MPs and the SLEDAI score (r = -0.46, *P *= 0.056). For RA patients, level of leukocyte-derived MPs and the DAS28 showed a significant negative correlation (r = -0.6, *P *= 0.005; Figure 4e).

Patients with AIDs receiving (n = 52) or not receiving (n = 37) immunosuppressive drugs or biological agents did not differ in level of MPs (total MPs: 8.4 ± 0.9 vs 7.1 ± 1.0, *P *= 0.13).

### Consumption of MPs by soluble PLA2

**Figure 5:**
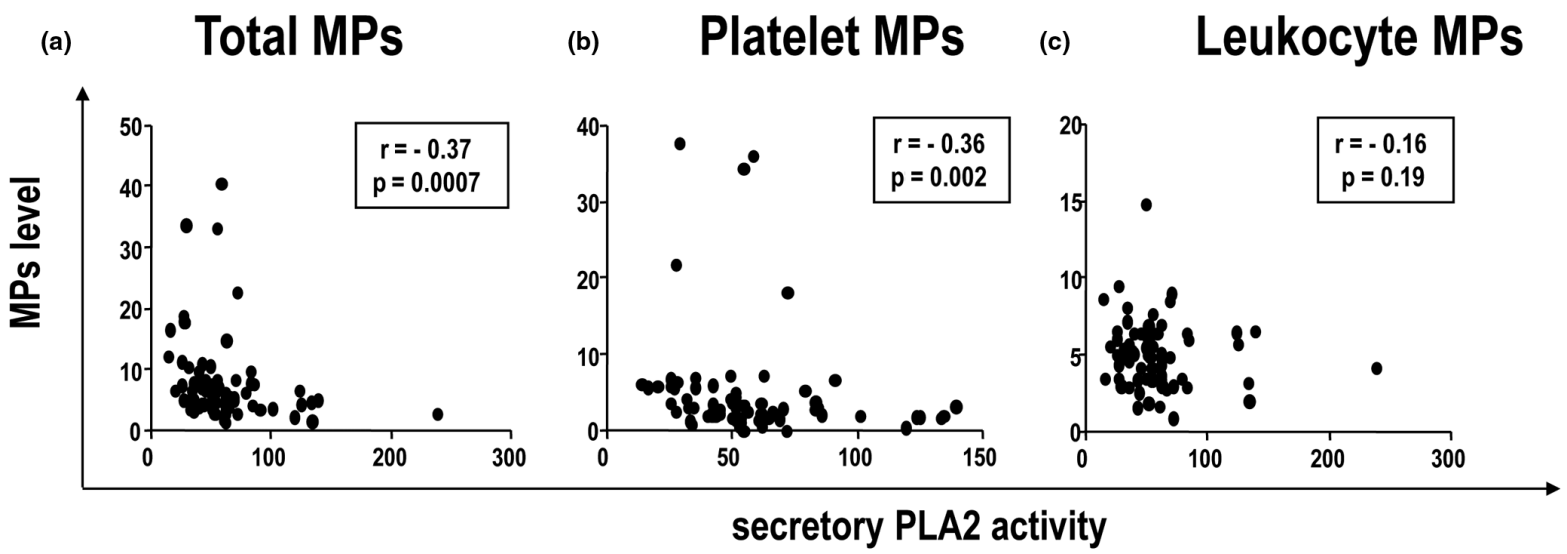
Correlation between plasma activity of secretory phospholipase A2 (sPLA2a) (expressed as nmol/min/mL) and plasma level of circulating **(a)** total microparticles (MPs), **(b)** platelet-derived MPs and **(c)** leukocyte-derived MPs (nM PS Eq).

We hypothesized that because MPs contain accessible anionic phospholipids such as PS, they could be consumed by sPLA2. This enzyme is increased in level and activity in some inflammatory diseases and catalyzes hydrolysis of aminophospholipids, including PS, as well as phosphatidylcholine and phsophatidylethanolamine, all contained in microvesicles [16]. Plasma sPLA2 activity was significantly higher in patients with pSS (*P *= 0.028), SLE (*P *= 0.036), and RA (*P *= 0.005) than in HCs (Table 2). Interestingly, the level of total MPs and activity of sPLA2 showed a significant inverse correlation for all patients with AIDs (r = -0.37, *P *= 0.0007 and r = -0.36, *P *= 0.002 for total and platelet MPs, respectively; Figure 5). Moreover, sPLA2 activity was significantly higher in the 14 pSS patients with extra-glandular involvement than in those with only glandular involvement (56.7 ± 3 versus 47.3 ± 5.2 nM/min/mL, *P *= 0.01). Conversely, MP level was not correlated with level of C-reactive protein, a classical marker of systemic inflammation.

### Increased levels of platelet activation biomarkers (sCD40L and sCD62P) in AIDs

As we found increased levels of platelet-derived MPs in the three studied AIDs and because platelet activation has been poorly assessed in pSS, we assessed plasma levels of sCD40L and sCD62P, two biomarkers of platelet activation (Table 2). The concentration of sCD40L was significantly higher in pSS and RA patients than in HCs and higher, but not significantly, in SLE patients than in HCs (*P *= 0.006, *P *< 0.0001 and *P *= 0.09, respectively). sCD62P levels were significantly higher in patients with pSS, RA and SLE than in HCs (*P *< 0.0001, *P *= 0.0003 and *P *< 0.0001, respectively). We found no association or correlation of level of these biomarkers with disease activity.

## Discussion

In the present study, we investigated the plasma level of circulating MPs in patients with the AIDs pSS, SLE and RA and found a higher level of total and platelet-derived circulating MPs as compared with HCs. A specific feature of pSS was an elevated level of leukocyte-derived MPs, which was not observed in other AIDs. Interestingly, in severe pSS with extraglandular manifestations, the level of platelet MP was less increased than those in pSS patients with glandular involvement only. In addition, we found an inverse correlation between level of MPs and disease activity in RA and SLE. Moreover, we found the level of MPs inversely correlated with two other quantitative biomarkers, serum β2 microglobulin level in pSS and anti-dsDNA IgG antibodies in SLE.

The patients in our study were slightly older than those in the HC group. No correlation has been observed between the total MPs levels and the age of patients in each disease group (*P *> 0.1). Likewise, no data in the literature suggest any impact of age on MP levels except in subjects younger than 18 years old [19]. The relatively small number of men in each group may probably not have an impact on MP levels: the comparison of the MP levels between men and women with AIDs has shown no difference according to the sex for each subtype of MPs (*P *> 0.16). To avoid the confounding effects of other factors susceptible to increase the level of MPs, such as cardiovascular risk factors or infection [20], we verified that associated cardiovascular co-morbidities might not have influenced the increased number of MPs. In addition, we have not included patients with recent thrombosis, acute or chronic infection who represent confounding factors disturbing the interpretation of results in AIDs. Finally, some patients have very high levels of MPs, suggesting that MP levels may be heterogenous in a defined disease group. However, after excluding patients with total and platelet MP levels above 20 nM PS Eq and leukocyte MP levels above 10 nM PS Eq in all groups, the statistical analysis remained unchanged (data not shown).

Circulating MPs originate from cell plasma membranes and are generated after cell stimulation (apoptosis or activation). In AIDs, MPs could be released at a systemic level by cytokine stimulation according to the same mechanism demonstrated *in vitro *[14,21,22]. The increase in the level of platelet MPs suggests that platelets were activated in the three diseases we studied. To confirm this feature in AIDs, the plasma concentrations of sCD40L and sCD62P, which are released by platelets upon stimulation and considered the two typical biomarkers of platelet activation [23], were increased in all AID groups as compared with HCs. This increase has been reported for RA and SLE [3,24,25], whereas in pSS, sCD40L has been reported only once [4], and sCD62P measurement in pSS has never been assessed. These data emphasize the known role of platelets in RA and SLE. Of note, activated platelets in SLE could activate plasmacytoid dendritic cells for interferon-alpha production [26]. These latter cells are also detected in labial salivary glands of patients with pSS [27]; hence, platelets could also contribute to plasmacytoid dendritic-cell activation in pSS.

As MPs can be detected by several non-standardized methods [28], we assessed MPs with two different methods simultaneously, solid-phase capture assay and flow cytometry, the results being positively correlated. Of interest, capture assay detects leukocytes and platelet MPs as being annexin V positive, whereas quantification of these subtypes of MPs by flow cytometry does not use annexin V ligation and thus involves annexin V-positive MPs as well as the small fraction of annexin V-negative MPs [2]. However, no clinical association with results obtained on flow cytometry was tested because few patients were tested with this method. Furthermore, tissue factor-positive MPs were not assessed in this study because of the low frequency of thombotic manifestations in pSS.

As MPs are generated after cell activation and/or apoptosis, it is not possible to discriminate between these two mechanisms to explain the increase in MPs in AIDs. If apoptosis play a role, it is probably not linked to immunosuppressive agents because the patients treated with these drugs did not have higher levels of MPs.

Increased plasma MP levels have been reported in metabolic, cardiovascular, infectious, neoplastic and autoimmune diseases [29]. In autoimmune diseases, MPs have been found elevated in RA [3,30], SLE [14,31], Crohn's disease [32], systemic sclerosis [22], vasculitis [33-35] and myositis [36]. Here we report the first assessment of circulating MPs in pSS. An interesting finding was the significantly decreased level of platelet MPs in pSS patients with more severe disease corresponding to extra-glandular involvement compared with those with glandular involvement only. A similar feature was also shown in patients with more severe SLE and RA disease as assessed by the SLEDAI and DAS28, respectively. However, in the three AIDs, the level of MPs in patients with more severe disease remained greater than in HCs. In fact, similar results have been recently reported in systemic sclerosis [22] and Crohn's disease [32] on assessment of MPs by flow cytometry and solid-phase capture assay, respectively. These results and the present results suggest that the level of circulating MPs might be inversely related to severity of disease as a general biological mechanism. In RA, discordant results have been reported: MP level was found increased or not different from that in HCs [30,37,38]. Finally, for other acute inflammatory diseases such as severe sepsis or multiple organ dysfunction syndrome, the number of platelet and endothelial MPs was found to be lower than that for controls [39] and a low level of MPs in severe sepsis was associated with a poorer prognosis [40].

Several hypotheses could explain these discordant findings. First, the decreased plasma level of MPs could be a result of consumption or confinement of MPs by adhesion in the tissue target of the AID such as the synovium in RA [41]. Second, MPs can aggregate circulating leukocytes and platelets, thus leading to the formation of leukocyte-platelet complexes. Thus, MP measurements do not take these MPs sequestered in cell aggregates into account, which leads to an underestimation of their amount [3,42]. These aggregates were found in higher levels in SLE and RA patients than in controls, but no association with disease activity has been reported to date [3,25,43]. Finally, the decreased level of MPs in active disease could be explained by the destruction of circulating MPs in the peripheral blood by phospholipases, especially sPLA2, which targets its aminophospholipid substrates in shedded membrane particles to generate lysophosphatidic acid [16].

Interestingly, we found a significant inverse correlation between levels of total MPs or platelet-derived MPs and sPLA2 activity in patients. We hypothesised that plasma MPs could be destroyed by increased sPLA2 through the degradation of their aminophospholipids in active disease. Thus, previous *in vitro *experiments showed that cell-derived microvesicles provide a preferential substrate for sPLA2 by the transformation of phospholipids present in MPs into lysophosphatic acid [16]. New experiments assessing a direct consumption of MPs by sPLA2 would be very interesting to perform.

sPLA2 activity was increased in all patients, especially pSS patients with extra-glandular involvement who showed a significantly decreased level of platelet MPs. Furthermore, although high level of sPLA2 has been reported in RA [44,45], we report for the first time in pSS and SLE the increased functional activity of sPLA2, despite the absence of increased levels of other classical biological markers of systemic inflammation (C-reactive protein and fibrinogen; Table 1). Thus, the exact role of sPLA2 in AIDs, in addition to its pro-inflammatory role, remains to be elucidated, especially in the context of cardiovascular complications observed in these diseases. Of note, we did not use a quantitative but rather a functional assay of sPLA2, which may better explain MP destruction in case of active disease.

To date, plasma level of MP has been considered a biomarker reflecting cell activation and could participate in the accelerated atherosclerosis observed in AIDs, but involvement of MPs in the cross-talk between resident cells in target organs of autoimmunity and inflammatory infiltrating cells has been sparsely reported. In RA, leukocyte MPs can activate synovial fibroblasts [21,46,47], but no data are available for pSS and SLE. Only exosomes, another kind of circulating vesicles containing specific auto-antigens and generated by salivary gland epithelial cells, have been identified [48]. As we found elevated MP level in pSS, the functional role of MPs remains to be elucidated, as does the role of platelet activation, despite the absence of increased thrombosis in this disease.

## Conclusions

We demonstrate that the level of circulating MPs is significantly elevated in pSS, as well as in RA and SLE, and could represent a new biomarker reflecting the systemic state of cell activation in these diseases. However, because the level of MPs increases less in patients with more severe disease, the interest of using MP levels for monitoring disease activity is limited, unless assessment of sPLA2 activity is performed in parallel. Indeed, a decrease in active disease could be related to a degradation process of MPs by sPLA2. Additional studies of function are needed to understand the involvement of MPs in signalling pathways of remote cellular cross-talk in AIDs and how platelets are precisely involved in pSS. Finally, investigation of the production of MPs at a local level in the target organs of autoimmunity, such as salivary glands in pSS, could be helpful for better understanding the role of these vesicles as mediators of the intercellular cross-talk.

## Abbreviations

AIDs: autoimmune diseases; APLS: anti-phospholipid syndrome; DAS28: Disease Activity Score 28; dsDNA: double stranded DNA; ELISA: enzyme-linked immunosorbent assay; GPIb: glycoprotein Ib; HC: healthy controls; Ig: immunoglobulin; mAbs: monoclonal antibodies; MGUS: monoclonal gammopathy of undetermined significance; MPs: microparticles; PS: phosphatidylserine; pSS: primary Sjogren's syndrome; RA: rheumatoid arthritis; sCD40L: soluble CD40 ligand; sCD62P: soluble P-selectin; SLE: systemic lupus erythematosus; SLEDAI: Systemic Lupus Erythematosus Disease Activity; sPLA2: secretory phospholipase A2; TNF: tumor necrosis factor.

## Competing interests

The authors declare that they have no competing interests.

## Authors' contributions

JS, VP, XM, and JMF were responsible for the study design, manuscript preparation, interpretation of the data and statistical analysis. JS, VP, and CM-R were responsible for sample blood collection. JMF, and FT were responsible for capture assay. JEG and JS carried out the statistical analysis. JS, VP, AJ, and SG contributed to flow cytometry experiments. MI, and JS performed ELISA experiments. JB, and JS performed sPLA2 activity measurements. All authors reviewed and approved the final manuscript.
